# Planning long lasting insecticide treated net campaigns: should households’ existing nets be taken into account?

**DOI:** 10.1186/1756-3305-6-174

**Published:** 2013-06-14

**Authors:** Joshua Yukich, Adam Bennett, Joseph Keating, Rudy K Yukich, Matt Lynch, Thomas P Eisele, Kate Kolaczinski

**Affiliations:** 1 Center for Applied Malaria Research and Evaluation, Department of Global Health Systems and Development, Tulane University School of Public Health and Tropical Medicine, 1440 Canal St., Suite 2200-TB46, New Orleans, LA 70112, USA; 2 Sensorstar, Inc., 787 Oella Avenue, Ellicott City, MD 21043, USA; 3 Center for Communication Programs, Johns Hopkins Bloomberg School of Public Health, 111 Market Place Suite 310, Baltimore, MD 21202, USA; 4 Chemin des Grands-Champs 5, 1279 Bogis-Bossey, Vaud, Switzerland

**Keywords:** Bed net, Long lasting insecticide treated bed net (LLIN), Malaria, Coverage, Mass campaign, Cost savings

## Abstract

**Background:**

Mass distribution of long-lasting insecticide treated bed nets (LLINs) has led to large increases in LLIN coverage in many African countries. As LLIN ownership levels increase, planners of future mass distributions face the challenge of deciding whether to ignore the nets already owned by households or to take these into account and attempt to target individuals or households without nets. Taking existing nets into account would reduce commodity costs but require more sophisticated, and potentially more costly, distribution procedures. The decision may also have implications for the average age of nets in use and therefore on the maintenance of universal LLIN coverage over time.

**Methods:**

A stochastic simulation model based on the NetCALC algorithm was used to determine the scenarios under which it would be cost saving to take existing nets into account, and the potential effects of doing so on the age profile of LLINs owned. The model accounted for variability in timing of distributions, concomitant use of continuous distribution systems, population growth, sampling error in pre-campaign coverage surveys, variable net ‘decay’ parameters and other factors including the feasibility and accuracy of identifying existing nets in the field.

**Results:**

Results indicate that (i) where pre-campaign coverage is around 40% (of households owning at least 1 LLIN), accounting for existing nets in the campaign will have little effect on the mean age of the net population and (ii) even at pre-campaign coverage levels above 40%, an approach that reduces LLIN distribution requirements by taking existing nets into account may have only a small chance of being cost-saving overall, depending largely on the feasibility of identifying nets in the field. Based on existing literature the epidemiological implications of such a strategy is likely to vary by transmission setting, and the risks of leaving older nets in the field when accounting for existing nets must be considered.

**Conclusions:**

Where pre-campaign coverage levels established by a household survey are below 40% we recommend that planners do not take such LLINs into account and instead plan a blanket mass distribution. At pre-campaign coverage levels above 40%, campaign planners should make explicit consideration of the cost and feasibility of accounting for existing LLINs before planning blanket mass distributions. Planners should also consider restricting the coverage estimates used for this decision to only include nets under two years of age in order to ensure that old and damaged nets do not compose too large a fraction of existing net coverage.

## Background

In recent years there has been a large scale-up of vector control for protection against malaria in sub-Saharan Africa (SSA), primarily through the expanded provision of insecticide treated bed nets (ITNs) and long lasting insecticide treated bed nets (LLINs) with funding from international sources [1]. Whereas previously, distributions of ITNs and LLINs have often been targeted towards pregnant women and young children, increasingly LLIN distribution campaigns aim to achieve high levels of ownership among the entire population. It is hoped that achieving high levels of ownership in the entire population, or universal coverage, will lead to reductions in malaria transmission at the community level [2-4], in addition to the personal protection offered to those sleeping under LLINs. As a result of recent scale-up efforts, many African countries have already achieved high levels of LLIN ownership [5-7] and reductions in malaria transmission [8-10].

LLINs, like most goods, do not last forever; nets are subject to wear and tear, deterioration of insecticidal effect, and loss [11,12]. Countries that have achieved partial or near universal levels of LLIN ownership will therefore need to continue distributions in order to sustain these levels [13]. Most countries also have a policy of continuous distribution of LLINs to maintain coverage through ante-natal care and immunization services or subsidized private sector sales; however, many of these systems are either not adequate to maintain LLIN coverage at universal levels or in some cases functional in name only. Given this, additional mass campaign distributions are likely to be conducted in the context of already existing LLIN coverage.

Mass distribution campaigns are often used to rapidly increase LLIN ownership and can be conducted as ‘blanket’ or ‘full’ campaigns, where every household is provided with the total number of nets necessary to meet universal coverage targets, or as ‘top-up’ campaigns, where existing nets in households are taken into account and each household is given only the additional number of nets needed to bring them up to the target number. The most obvious benefit of taking into account existing LLINs during ‘top-up’ campaigns is the potential for cost saving. With LLINs costing approximately $4 each [14], funding may be wasted if ‘blanket’ distributions give a full quota of new LLINs to every family, regardless of how many they already have. *A priori*, as malaria control resources are scarce, this seems like a desirable strategy. However, in reality the decision to account for existing nets will be influenced by more than pure commodity cost differences, as consideration must be given to whether cost-savings will be achieved after factoring in additional distribution costs, and whether the approach is appropriate in terms of equity and health outcomes.

This paper aims to stimulate discussion around whether existing nets should be accounted for during follow-up mass distribution campaigns. A second aim focuses on generating simulations to provide planners with evidence to help decide as to when and where nets should be accounted for under programmatic conditions. This requires planners to be able to estimate existing LLIN coverage, expected cost-savings, and the epidemiological implications of their decisions; methods for making these estimates are presented and discussed.

## Methods

**Figure 1:**
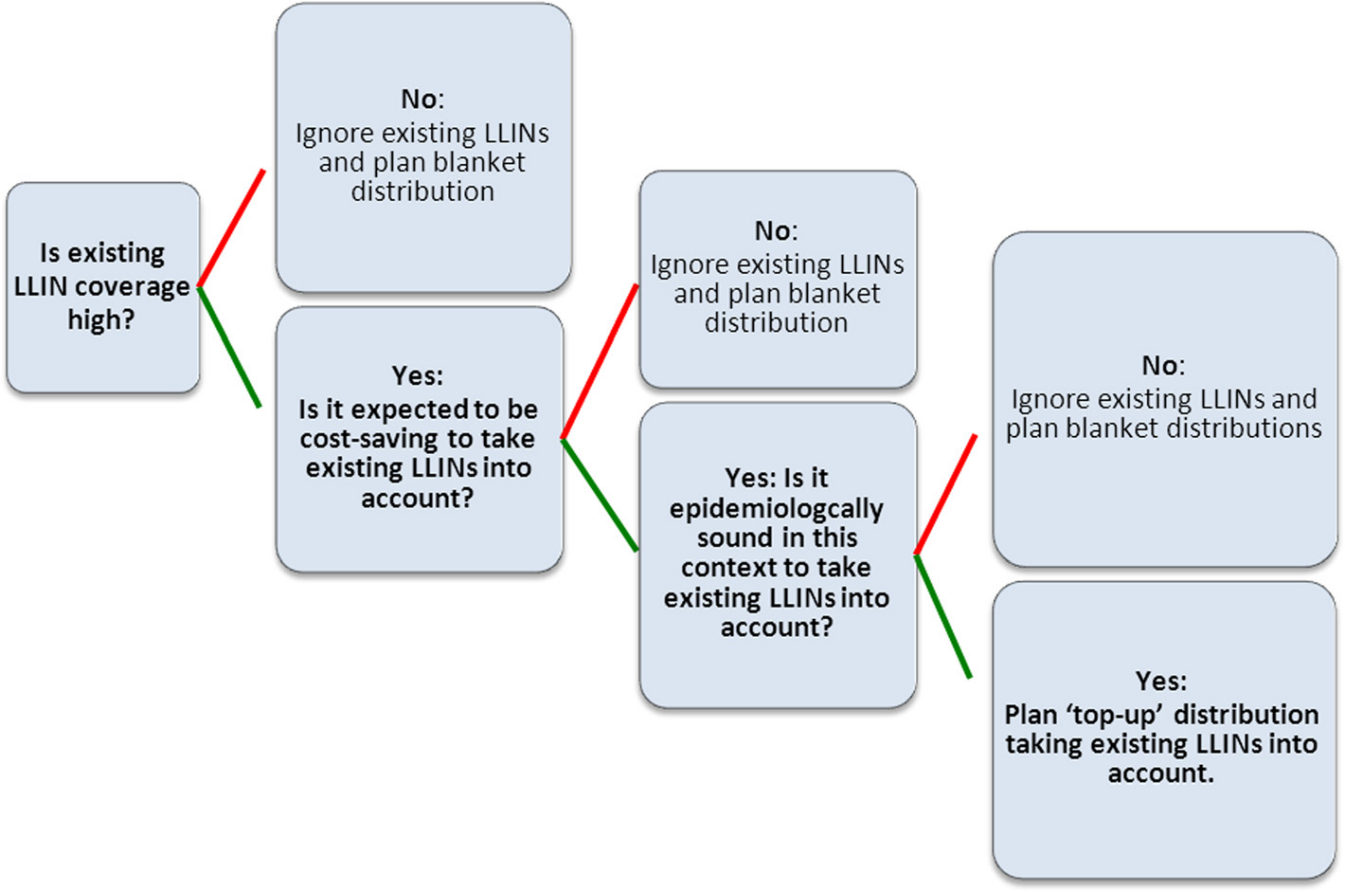
Decision tree outlining key inputs for determining whether to account for existing nets or not.

Deciding whether or not to take existing nets into account in any given setting requires planners to be able to answer a few basic questions about the setting in which the campaign is expected to occur (Figure 1).Unfortunately, how best to answer these questions is not obvious. Here, two approaches are used; the first uses simple mathematical and cost modeling of coverage and costs of LLIN distribution campaigns in a stochastic simulation format, and the second relies on review of existing peer-reviewed literature to provide guidance. Below is a description of which methods are applied to each specific decision point, as well as any sub-questions addressed.

### Determining existing LLIN coverage

The first question in the decision tree in Figure 1 requires determining whether existing LLIN coverage is sufficiently high to make accounting for existing LLINs worthwhile. For the purpose of this manuscript we have used household survey data to establish LLIN coverage, defined as the number of households owning at least one LLIN. The average number of LLINs of a given age per household and the over-dispersion of LLIN counts at the household level were the principle model parameters used to estimate coverage. The over-dispersion parameter describes the relationship between the mean and variance for count data; high over-dispersion indicates that the variance is higher than would be expected given the mean if the data were Poisson distributed. Both parameters are directly measureable using demographic and health survey (DHS) and malaria indicator survey (MIS) data. This approach allows decision makers to include the distribution, coverage and age of existing LLINs, the uncertainty of survey estimates, and the timing of the survey relative to a planned distribution (which will affect estimates of current coverage) into the decision making framework.

We developed a model based on the NetCALC software [15], a spreadsheet model, to estimate LLIN coverage and uncertainty. NetCALC is a simple deterministic cohort model that uses the timing and size of a net distribution, population and household size, population growth, and net decay to estimate household net coverage levels over time. To capture decision making around planning a “top-up” campaign in the context of existing LLIN coverage, we modified the NetCALC model to incorporate information on existing coverage obtained from survey data and implemented the model in [R] [16].

Code and demonstration of how to use the [R] software to conduct simulations are included here as Additional files 1 and 2. Additional file 1 contains instructions for the installation and use of the software, while Additional file 2 contains the model code in the form of an [R] package NetCalc_0.2.tar.gz, which can be installed and run in an [R] environment. Additional file 3: Figure S2 is an illustrative example of a simple use of the software.

This modification allowed for the input of a vector containing the estimated average number of LLINs per household of a given age that were present at the time of the survey. To simulate the effect of sampling error on the decision of whether to account for LLINs in planning a “top-up” campaign, we modeled the total number of existing nets by sampling from a negative binomial distribution of the number of nets of a given age owned by households at the time of the survey. Data from thirty-three MIS and DHS surveys were used to generate estimates of the over-dispersion parameter for the model. Over-dispersion parameters estimated from MIS and DHS data ranged from below one to approximately 14, with most parameters at moderate or high coverage close to one. This indicates that while the distribution of nets among households tends to follow a Poisson distribution at higher coverage, at lower coverage the negative binomial distribution is more appropriate (See Additional file 4 for details). The over-dispersion parameter for the estimated average number of LLINs available per household in each survey country was determined to be near to one in the coverage ranges of greatest interest for purposes of this paper (moderate coverage *e.g.* between 20% of households owning at least one LLIN and 70-80% of households owning at least one LLIN) (See Additional file 5: Figure S1 and Additional file 4). These simulations resulted in vectors of the numbers of nets available in each survey and confidence bounds, and these results were used as inputs in model simulations.

Time since a survey may also impact decisions around existing coverage levels in two ways. First, nets decay, are lost, disposed of, repurposed or otherwise cease to exist over time. We accounted for this by allowing survey data to be used to measure the age of existing nets, the number of existing nets and the time that was expected to pass before the distribution of interest was simulated. Lifetimes of LLINs were modeled using the “smooth-compact” decay function fit by Nakul Chitnis to Albert Killian’s LLIN retention data [17].

Second, current expected coverage after a survey can be affected by distributions of LLINs between the survey estimate and the time of the planned mass distribution. We incorporated these estimates by imputing new LLINs into the simulation as described below. We only considered routine distribution systems here as it was assumed that such a decision around whether or not to account for existing LLINs would be at the time of the first mass distribution after a coverage survey, rather than many years or many mass distributions later. As many African countries have used existing antenatal care (ANC) and expanded program on immunization (EPI) platforms to distribute LLINs, we based our routine net distributions in simulations accordingly.

We simulated varying scales of LLIN mass distribution using multiple stochastic simulations of net decay and initial coverage while varying the estimated initial coverage from an average of zero nets per household to 3 nets per household and the routine distribution system from 0% coverage with ANC and EPI to 100% coverage with both; 1,000 simulations were conducted for each scenario and an average household size of 5.5 persons per household (details in the Additional file 4). These ‘Monte Carlo’ simulations allowed us to determine the fraction of simulations where a coverage target was achieved when a survey coverage estimate was combined with the size of a mass distribution campaign and a level of routine distribution [18]. We also varied the length of time between surveys and mass distributions over a period of one to three years to account for decay of nets after the coverage survey was conducted. All simulations assumed an initial population size of 1,000,000 and an annual growth rate of 3% [19].

### Determining expected cost-saving when taking existing nets into account

The second question in the decision tree is whether or not cost-savings would be expected if planners decide to take existing LLINs into account when planning future LLIN distributions. Deciding whether or not it makes financial or economic sense to account for existing nets requires a tradeoff. If existing nets are not accounted for in the context of a mass distribution campaign, the number of LLINs required to achieve a set coverage target should be estimated as if no LLINs were already present. When existing LLINs are accounted for, the number of LLINs delivered will be reduced by the number of LLINs of useable quality expected to be found in the target area. The tradeoff is in the expectation that distributing nets while accounting for existing nets will be more costly (per LLIN) than simple blanket mass distribution. Thus, whether accounting for nets is sensible in a financial sense will depend on the existing number of LLINs in the community, the distribution cost (per LLIN) when not accounting for existing nets, and the distribution cost (per LLIN) when accounting for existing LLINs.

The overall cost of a campaign can be calculated by multiplying the number of LLINs distributed (*N*_*o*_) by the cost per LLIN of the net and distribution (*C*_*o*_). Here we use the subscript *0* to represent a campaign which does not account for existing nets and the subscript *A* to denote a campaign which does. There exists a point at which the tradeoff between savings from distributing reduced numbers of nets in a campaign that accounts for existing nets and the increased per net cost of this distribution will be equal. This can be represented by the following equation:

$$C_{0}N_{0}=C_{A}N_{A}$$

At this point there will be no cost savings from trying to account for nets. This equation can be rearranged to illustrate the ratio between costs or net numbers where this point occurs, this is called the break-even point (*R*_*I*_).

$$\frac{C_{A}}{C_{o}}=\frac{N_{o}}{N_{A}}=R_{I}$$

Where *C*_*o*_ is equal to the cost per net of distributing nets without accounting for existing LLINs, *C*_*A*_ is equal to the cost per net of distributing nets while accounting for existing LLINs, *N*_*o*_ is the number of LLINs required for reaching the coverage target when existing nets are not taken into account and *N*_*A*_ is the number of LLINs required to reach the target coverage when accounting for existing LLINs in a setting in which all existing nets can be properly identified in the field.

Given this relationship, when a program estimates that accounting for LLINs vs. not accounting for LLINs will yield a cost ratio greater than *R*_*I*_*,* it will be more costly to account for existing LLINs than to make a blanket distribution. Alternatively, for ratios less than *R*_*I*_ it will be cost saving to account for existing LLINs.

Under programmatic conditions, it is expected that the feasibility of identifying existing nets in houses may be less than perfect, likely leading to an underestimation of the true number of existing useful nets. This may be due to household respondents not declaring nets that they actually own in order to get new nets, or failure of enumerators to accurately identify all useful nets at the time of the household registration for the distribution campaign. Because of these potential failures, more nets need to be distributed than under ideal conditions of perfect identification of useful nets in pre-distribution registration. We describe the level of incomplete net identification by the proportion of nets that actually exist which are recorded during the household registration and call this proportion *F*. When this proportion is incorporated into the previous relationship, we can construct a new ratio, which is the break-even point under conditions of imperfect identification of nets in the field (*R*_*F*_). The expression below shows the break-even point for ratios of costs in the context of having to distribute more nets than actually needed based on coverage levels, as a result of the incomplete registration process.

$$R_{F}=\frac{N_{o}}{N_{A}+\left[\left(N_{o}-N_{A}\right)\;\left(1-F\right)\right]}$$

If combined with *R*_*I*_ this relationship can be rewritten as follows in terms of only *R*_*I*_ and *F*.

$$R_{F}=\frac{R_{I}}{R_{I}-R_{I}F+F}$$

It is also important to define the cost ratio (per LLIN delivered) of a campaign that accounts for LLINs relative to a campaign that doesn’t in terms of the cost of LLINs (assumed to be the same in either scenario), and the cost of distribution (assumed to vary between the two scenarios). This is necessary to examine the effect that changes in the distribution cost may have on the overall ratio of the costs of each campaign (*R*_*C*_).

$$R_{c}=\frac{C_{N}+R_{A}C_{D_{o}}}{C_{N}+C_{D_{o}}}$$

In the above equation, *R*_*c*_ is the ratio of the cost (per LLIN) of a campaign that accounts for LLIN to the cost (per LLIN) of a campaign that does not, *C*_*N*_ is the cost of a LLIN, $C_{D_{o}}$ is the cost of distribution per LLIN when not accounting for a LLIN and *R*_*A*_ is the ratio of distribution costs per LLIN when LLINs are accounted for as compared to when LLINs are not accounted for. From the above equation it can be seen that *R*_*C*_ depends not only on the distribution cost when accounting for LLINs and not accounting for LLIN, but also on the relative size of distribution costs compared to LLIN costs. Additionally, where *R*_*C*_ is less than the break-even point considering the feasibility of accounting for LLINs in the field (*R*_*F*_), it would be desirable to try to account for LLINs; when *R*_*C*_ is higher than the break-even point accounting for LLINs would not be expected to be cost saving.

In the above equation there are two cost parameters, *C*_*N*_ and $C_{D_{o}}$. These parameters could be expressed in an alternative format as one cost parameter, *C*, representing the total campaign cost per LLIN when no accounting for existing nets is undertaken, and a unit-less parameter *B* which represents the proportion of commodity costs out of the total per LLIN cost, such that *C*_*N*_ = *BC* and $C_{D_{0}}=\left(1-B\right)C$. We can show that *R*_*C*_ depends only on *R*_*A*_ and *B* by means of substitution as demonstrated in the following two equations.

$$R_{C}=\frac{\mathrm{BC}+R_{A}\left(1-B\right)C}{\mathrm{BC}+\left(1-B\right)C}$$

This reduces to the following equation, which shows that the total cost of a campaign where existing LLINs are not accounted for has no direct effect on the ratio between the costs of a campaign that accounts for nets and one that does not.

$$R_{C}=B+R_{A}-BR_{A}$$

This equation implies that *R*_*C*_ can be estimated independently of the cost of an LLIN, as long as the procurement cost of an LLIN is not expected to be significantly different under the two scenarios. Further, it gives credence to the intuitive notion that what is important is the relative cost of the distribution component of the campaign costs in the accounting vs. not accounting scenarios, *R*_*A*_, and the contribution of this distribution cost to the total campaign costs per LLIN in the not accounting for existing nets scenario, *B*.

Finally, this leads to the conclusion that where *R*_*C*_ estimated for a specific setting is less than *R*_*I*_ or *R*_*F*_ (when feasibility is considered), accounting for existing LLINs would be considered to be cost-saving. The mathematics above can be applied very simply in real settings and only require that campaign planners estimate the distribution costs under the two alternative scenarios of accounting vs. not accounting, the feasibility of identifying nets in the field and the per LLIN procurement cost in order to determine if the campaign would be expected to be cost-saving.

### Epidemiological implications of accounting for nets versus not accounting for nets

The above methods detail the estimation of LLIN coverage and the costs of campaigns under various scenarios; however, understanding the epidemiological implications of accounting for existing LLINs in mass campaigns is somewhat more challenging. While LLINs have been shown to provide individual and community-level protection against biting mosquitoes and subsequent malaria morbidity and mortality across diverse settings [2,3,20-23], several important questions remain concerning when nets need replacement to maintain optimal protective effects. These include: How long do LLINs perform under programmatic conditions? Do older nets increase vector resistance? And, what is the optimal coverage needed to ensure a mass effect over time? Current estimates suggest the average useful life span of LLINs is 3 to 4 years, although weather conditions, materials used during production, washing habits, house and sleeping space type, physical conditions, and insecticidal efficacy likely influence net durability and effectiveness [23].

In order to address the epidemiological implications of accounting for existing LLINs, two lines of inquiry were followed: one was to model the average age of a ‘net crop’ in a population where campaign distributions accounted for nets and scenarios where campaigns did not account for existing nets. Secondly, a literature review was conducted to identify previous research relevant to the assessment of the epidemiological implications of accounting for existing nets versus not.

We modeled the average age of the net crop over time using a set of scenarios in the presence and absence of a routine distribution system. Four scenarios were simulated in the presence of an estimated 1.4 nets per household (HH) found during a recent coverage survey. This level represents a moderate level of household net ownership where it *might* be advantageous to account for existing nets. We simulated a single mass distribution two years after the coverage survey, where we either attempted to account for existing LLINs or did not attempt to account for nets, and where there was either a functional routine system in place or no functional routine system in place.

## Results and discussion

### Is Existing LLIN coverage high?

An example of the implementation of the model with accompanying [R] code is included in the web appendix to the paper, which illustrates how to run the simulations and a presentation of typical results.

LLINs present in a country are likely to have been distributed via multiple mechanisms, including routine ANC-EPI or other mass distribution campaigns. Furthermore, detailed information on LLIN coverage may be available from coverage surveys such as the MIS or DHS. Such surveys now contain net registries that require enumerators to list all nets present in the household as well as the reported age of observed nets. As such, they allow for the estimation of the existing numbers of nets in a country and the age structure of the existing net population (by year if the net is reported to be less than 36 months old).

**Figure 2:**
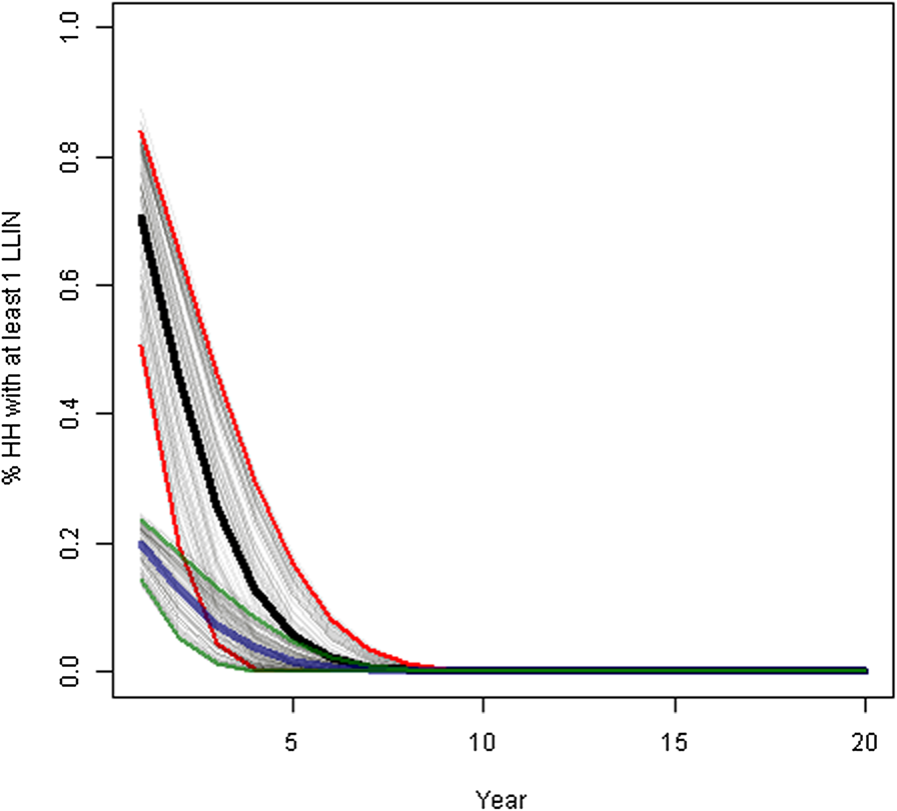
**Estimated household coverage of LLINs from the year following a simulated household survey.** Legend: Mean estimate of the percentage of households owning at least one LLIN derived from 1,000 simulations in the high coverage scenario are shown in black (95% of simulations from this scenario falling between red lines) mean estimates of the percentage of households owning at least one LLIN derived from 1,000 simulations for low coverage shown in blue, with 95% of simulations for this scenario falling between the green lines. Light grey lines represent values of individual simulation runs.

Figure 2 Illustrates the change in coverage over time from survey year forward, assuming an initial coverage of 100,000 LLINs distributed in the year of the survey and 100,000 LLINs that were one year old at the time of survey (or approximately 1.1 LLIN per household); we also used an initial coverage estimate of 200,000 LLINs that were new and 200,000 LLINs that were one year old (or approximately 2.2 LLIN per household). Surveys were simulated using a sample size of 3,000 households to represent a typical sample size for a DHS or MIS survey. Visual inspection of Figure 2 shows that the uncertainty derived from national level coverage estimates should affect the coverage estimation and that this uncertainty is projected forward in time, but eventually declines as projected coverage tends toward zero in the distant future. One year after a survey which yielded estimates of LLINs available of 100,000 new LLINs and 100,000 one year old LLINs, 95% of simulations of household ownership of at least one LLIN fell between approximately 18% and 25% (shown in blue and green in Figure 2). If the survey had resulted in estimates of 200,000 new LLINs and 200,000 one-year-old LLINs the coverage estimate in the current year was clearly even higher, but so was the range of possible estimates produced by the model (shown in black and red in Figure 2). These ranged in year two (two years after the survey) from approximately 50% to 80% for the indicator of household ownership of at least one LLIN. These scenarios are meant to be illustrative only and for all the other input parameters (*e.g.* total population, population growth rate, household size, over-dispersion) standard simulation parameters as described in Additional file 4 were used.

### Is it expected to be cost–saving to take existing LLINs into account?

**Figure 3:**
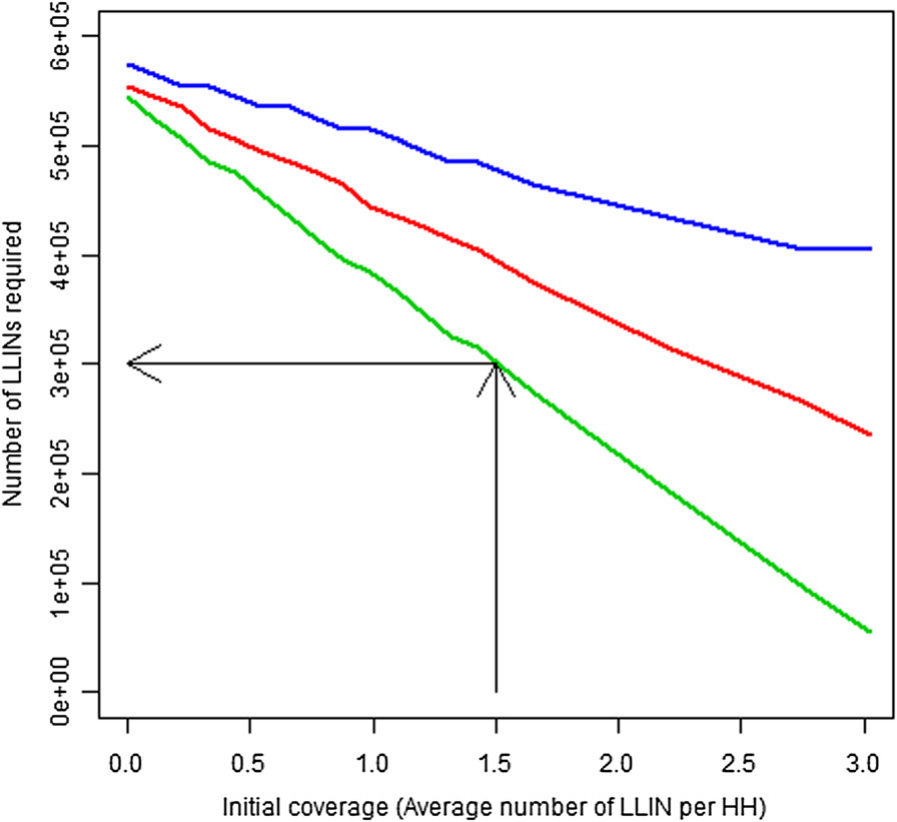
**Numbers of LLINs required to reach 90% coverage for varied initial coverage and times since survey.** Legend: This chart illustrates the numbers of LLINs that would be required to reach 90% household ownership of at least one LLIN in more than 80% of simulations for a given scenario with a mass campaign that accounts for existing LLINs at varied initial LLIN coverage levels and for campaigns conducted at varying times following the initial coverage survey. All simulations were conducted with a starting population of 1 million persons and an average household size of 5.5: Green line represents one year after the survey, red line two years after survey and blue line three years. One thousand simulations were conducted for each data point (combination of initial coverage estimate and delay until campaign). The black lines are intended as an aid to demonstrate interpretation (showing that for a campaign planned one year after the survey which showed an initial coverage of 1.5 LLIN per household the expected numbers of LLINs required in to reach a specified coverage level).

**Figure 4:**
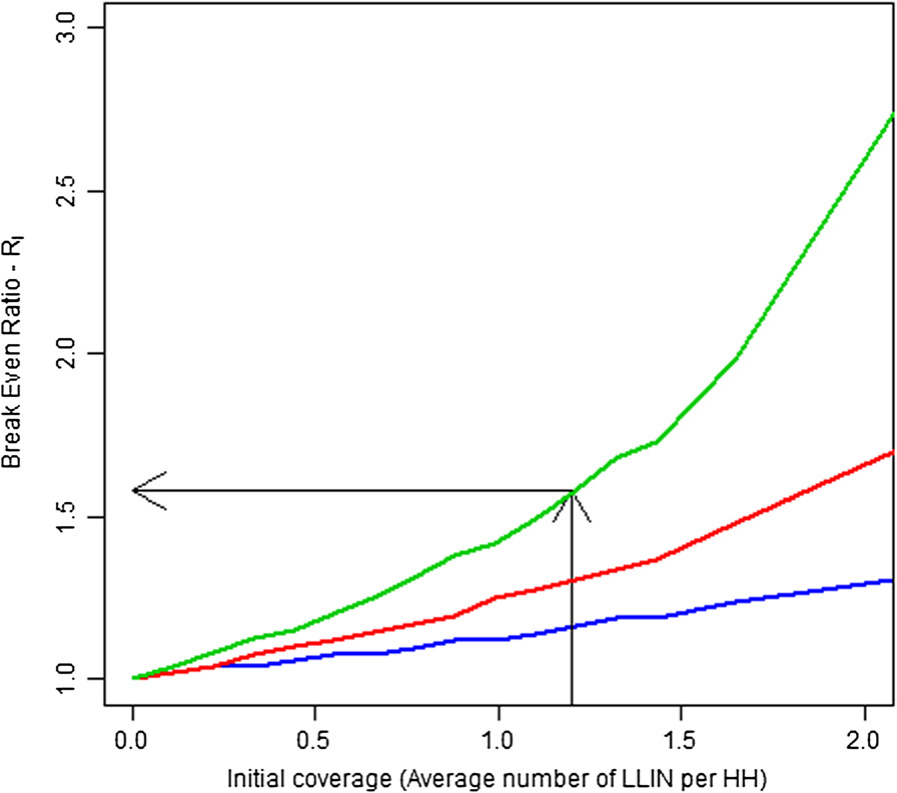
**Break even ratio under ideal conditions as initial coverage is varied.** Legend: Break even ratio is shown for three levels of length of time since the coverage survey was conducted. Survey one year prior shown in green, two years prior shown in red, and three years prior shown in blue.

Answering this question first required determining the number of nets that would be required to achieve a desired level of coverage under varying initial conditions of household coverage, net age and time between the survey and the planned mass-distribution. Figure 3 illustrates the relationship between initial coverage levels, time since the coverage survey and estimated net requirements. As time since the initial coverage survey increases, larger number of LLINs will be required to reach a desired coverage level. The black lines are intended as an aid to demonstrate interpretation (showing the expected numbers of LLINs required to reach a specified coverage level for a campaign planned one year after a survey that showed an initial coverage of 1.5 LLIN per household). A distribution that does not account for nets could be crudely thought of as requiring a horizontal line extending from the point at which each line intersects the y-axis though it will actually slope upward slightly due to population growth. Figure 4 shows various ratios of *R*_*I*_ (the break even ratio when all existing nets can be identified such that cost ratios lower than this would be expected to be cost saving) as initial coverage (average number of LLINs less than one-year-old from a survey) is varied for campaigns conducted one, two or three years after a coverage survey. This figure illustrates that as coverage increases it becomes more desirable to account for existing nets, thus the break even ratio or the ratio of costs below which it would be cost saving to account for existing nets increases, meaning that a campaign planner could expect to spend more money per LLIN to account for the existing nets and still expect to make a cost savings compared to a blanket distribution. As time since the survey increases the ratio falls, indicating that coverage has declined due to net decay, thus reducing the expected coverage level at the time of the campaign. The above figures are based on the assumption that no routine distribution system exists and as such no substantial influx of nets would be expected in the interim.

**Figure 5:**
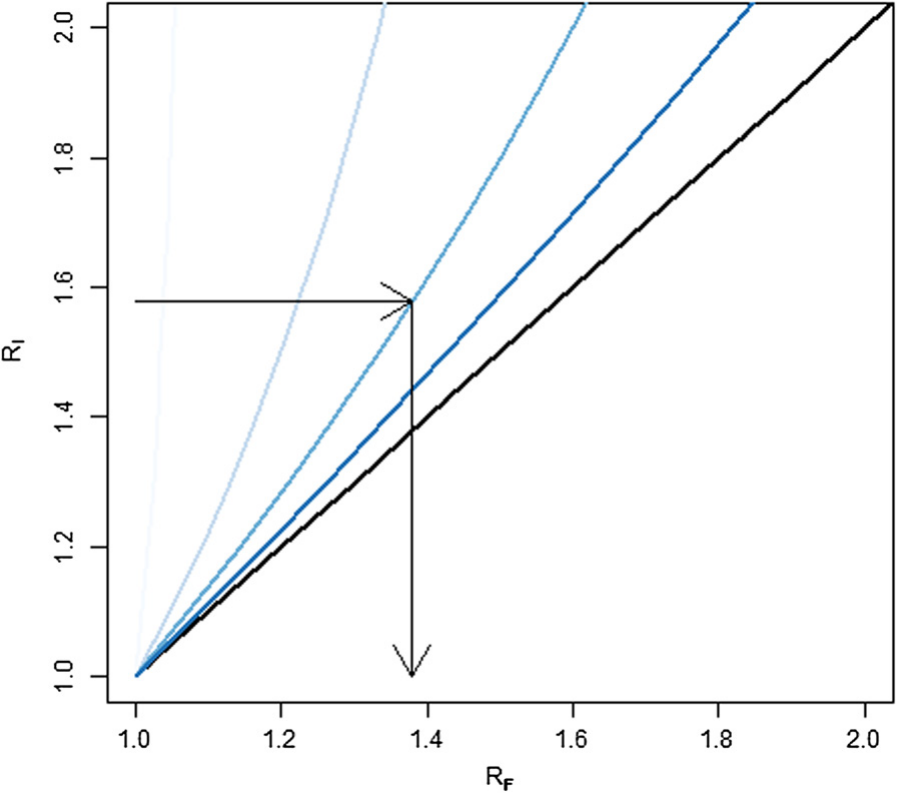
**The relationship between *R***_***F ***_**and *R***_***I***_**for varying levels of *F. *Legend: *F *(feasibility) of 10%, 50% 75% and 90% shown from light to dark.** Line in black shows ratio of one, *R*_*F*_ = *R*_*I*_, representing a scenario in which all nets are found *R*_*I*_ is the break even ratio under ideal conditions and *R*_*F*_ is the break even ratio when feasibility is considered. Arrows represent the relationship between an *R*_*I*_ and *R*_*F*_ when *R*_*I*_ = 1.6 and *F* = 0.75*.*

**Figure 6:**
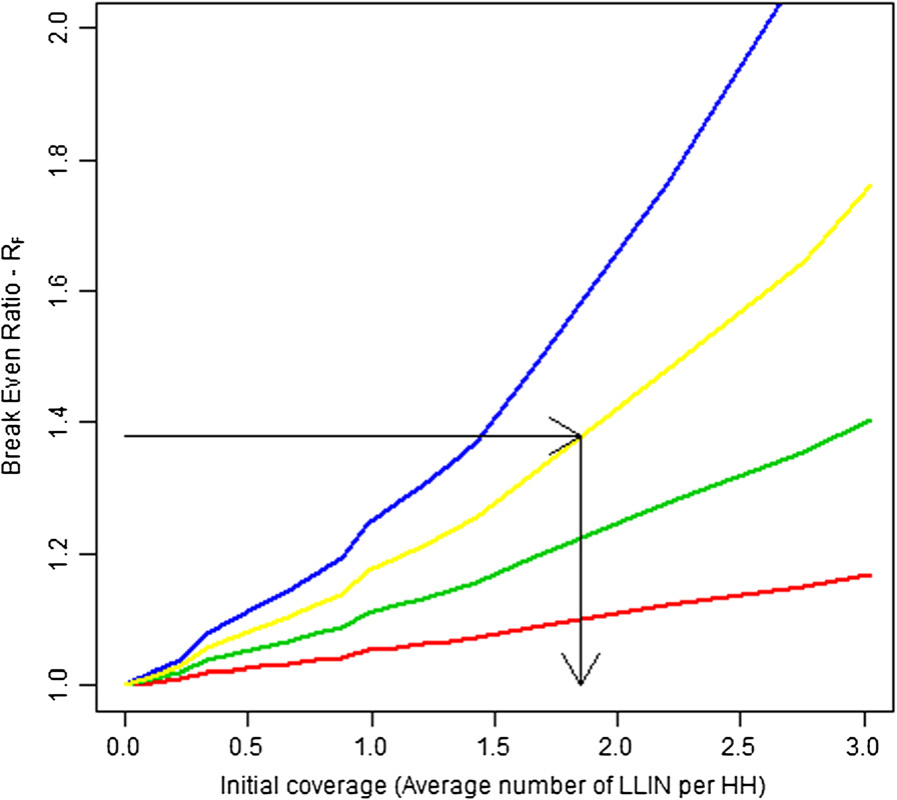
**Break-even ratios for a mass distribution after a survey with varying levels of initial coverage.** Legend: Mass distribution conducted two years after a coverage survey. Initial coverage defined in terms of average number of LLINs per HH breakeven ratios (*R*_*F*_ ) shown for varied levels of feasibility (*F*) of detecting LLINs in the field. The blue line represents ideal detection, yellow *F* = 0.75 detection, green *F* = 0.50, and red *F* = 0.25 detection. Arrows represent the *R*_*F*_ ≅ 1.4 and initial coverage to break even under *F* = 0.75 feasibility and a campaign conducted two years after the coverage survey with *R*_*F*_ ≅ 1.4 is approximately equivalent to *R*_*I*_ = 1.6 if *F* = 0.75 (See Figure 5).

Under conditions of less than ideal feasibility, *i.e.* when all existing nets cannot be accurately identified during campaign registration, this relationship is modified. The relationship between *R*_*I*_ and *R*_*F*_ is shown in Figure 5 for various levels of *F*. Figure 5 suggests that as *F* increases the ratio *R*_*F*_ tends towards one regardless of the initial level of *R*_*I*_. This implies that as the difficulty of identifying existing nets in the field increases, the rationale for doing so on a financial basis becomes less attractive. The implication of changing levels of *F* is that as feasibility declines, accounting for nets must become less expensive to be desirable (Figure 6).

Studies of the cost of distributing LLINs through campaigns or routine mechanisms are plentiful and have been the focus of several review articles and multi-country studies [14,24-26]. White and others conducted the most current and comprehensive review of LLIN cost, and they found a median financial cost per ITN distributed of $7.03 with a range from $2.97-$19.20. They estimated that approximately 63% of the costs were attributable to the costs of the net itself, with the remaining related to costs for various mechanisms used to deliver nets to households or to monitoring of campaigns. Other research has indicated that the delivery costs may be a smaller proportion of total costs and that total costs may be lower in the context of mass campaigns than in other routine systems [24,25]. Additionally White and colleagues found a median economic cost of $4.15 for distribution of nets with a range from $2.97-$10.05. After standardizing denominators into year of protection provided by a net they found a median estimate of $2.20 with a range of $0.88-$9.54 per year of protection. Nearly all of the studies reviewed above indicated that distributing LLINs in the form of routine distribution or mass campaigns is a cost-effective health intervention in low income settings with significant malaria risk.

No published peer-reviewed studies were identified that focused on determining the operational costs of accounting for existing LLINs in the field. Reviews of grey literature, and experiences from Cross-Rivers State in Nigeria, Senegal and a mass catch-up campaign in Tanzania indicated that accounting for nets in the field would significantly add to the cost of delivering nets on a per LLIN unit cost basis as compared to the mass “blanket” distribution that did not account for nets [27,28](H. Koenker; *unpublished data*). It is possible that the effect of accounting for nets on the proportion of the costs of a campaign related to LLIN distribution could be significant. However, as the largest proportion of total costs of a mass LLIN campaign are due to the actual LLIN procurement, the overall impact on estimated campaign costs is likely to be more limited. In the absence of large amounts of concrete data we examine here the implications of changes in distribution cost on the total cost of net distribution.

**Figure 7:**
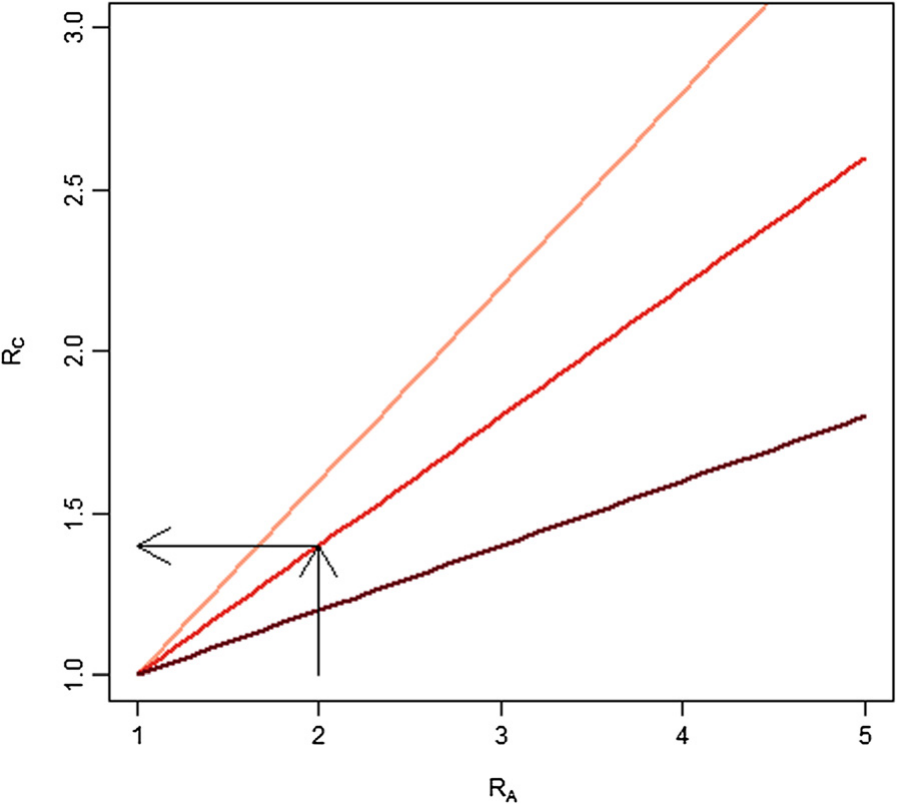
**Effect of *B *on the ratio of total costs when accounting vs. not accounting.** Legend: *R*_*a*_ (the ratio of distribution costs between a campaign which accounts for LLINs and one that does not) *vs. R*_*c*_ (the ratio of total per LLIN costs of a campaign that accounts for nets compared to one that does not) as *B* (the proportion of the per LLIN costs of the campaign which does not account for existing nets which is due to the commodity cost of the LLIN itself) is varied from 40% of the total costs for a blanket campaign to 80%. The lightest red line corresponds to *B* = 40% while the middle line corresponds to *B* = 60% and the darkest line corresponds to *B* = 80%. Arrows indicate how to read the relationship between a doubling of *R*_*a*_ and the effect of *R*_*c*_ for a level of *B* = 60%.

Figure 7 illustrates the implications of this definition of *R*_*C*_ for several plausible levels of relative costs of nets and distribution. This relationship demonstrates that the overall effect on campaign costs is minimized when LLINs commodity costs comprise the majority of total campaign costs. Evidence from past research indicates that 50%-70% of campaign costs are likely to consist of the LLIN commodity cost itself [14,25]. The red (middle) line, representative of 60%, is probably the most indicative of the relationship between the two in practice; when *B* = 0.6 a doubling in distribution costs is roughly equivalent to *R*_*C*_ = 1.4. This *R*_*C*_ value can be referenced to Figures 4 and 6 (see black lines on these figures) to determine whether the *R*_*C*_ value for this scenario would indicate that accounting for existing nets would be expected to break even or be cost saving in an ideal situation (Figure 4). Or whether the *R*_*C*_ value estimated would break even or be cost-saving under a more realistic scenario (Figure 6) where imperfect detection of existing nets is assumed. We illustrate this for a campaign planned two years after a coverage survey was conducted using the black lines on these charts. These results indicate that if the *R*_*C*_ value is estimated to be equal to 1.6 then under ideal conditions accounting for nets would be expected to be cost saving at an initial coverage level of above around 1.2 LLIN per household (~40% coverage of HH owning at least one LLIN) and under more realistic conditions (with *F* = 0.75) accounting for existing nets would be expected to be cost saving at an initial coverage level of 1.8 LLINs per household (~60% of HH owning at least one LLIN). This indicates that around 35-40% household ownership of at least one LLIN may be a reasonable point for programs to consider the financial and epidemiological implications of accounting for existing LLINs in a mass campaign when the campaign is expected to be conducted within the two years following the survey and feasibility of identifying nets in the field is high (near 100% or *F* = 1).

### Is it epidemiologically sound to withhold new LLINs where there are existing ones?

Our models have established that it is feasible and likely cost-saving to account for existing nets if feasibility of identification is at least 75% and household ownership of at least one LLIN is above 40%. Thus, we have also shown that we can achieve high and equal coverage with a distribution using either mechanism. The main factor that will then differ between the two scenarios (accounting vs. not accounting) is the age profile of the net crops. It is known that effective protection decreases over time due to both physical and chemical deterioration [29-32]; what is not known however, is the maximum net age before deterioration negatively impacts protective effects.

**Figure 8:**
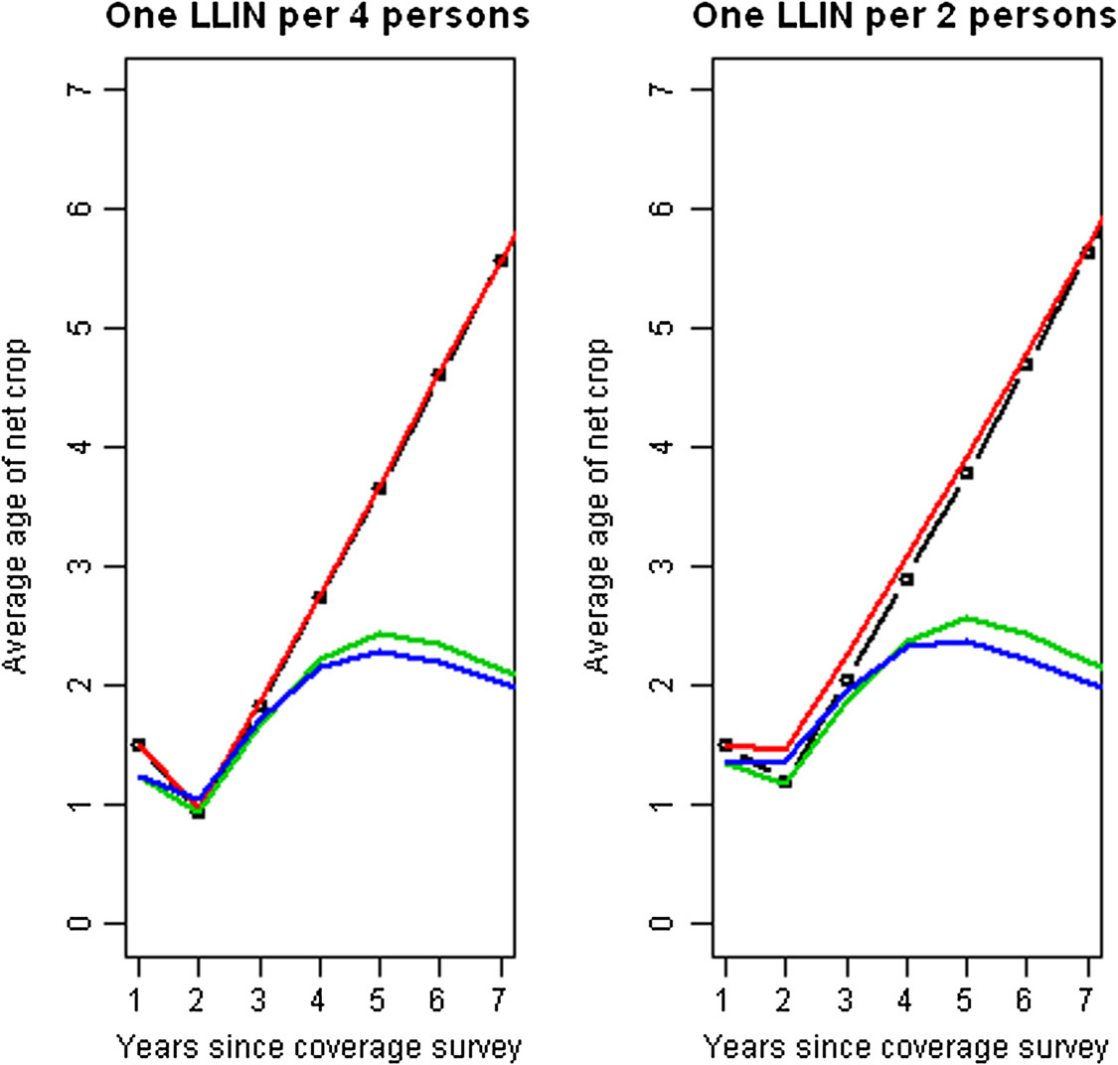
**Average age of net crop under different distribution scenarios and different initial coverage.** Legend: Black lines with circles show not accounting for existing LLINs with no routine system in place. Red lines show accounting for LLINs with no routine system in place. Green lines show not accounting for LLINs with a functional routine system in place and blue lines show accounting for LLINs with a functional routine system in place.

The simulations indicated that accounting vs. not accounting for nets made very little difference in the expected average age of the net crop. This result was largely insensitive to pre-existing coverage levels (i.e. one LLIN per two persons versus one LLIN per four persons), and was robust in the presence or absence of an existing routine distribution system. Age distributions of nets after two years post campaign were divergent in scenarios with routine distribution when compared to those without distribution. These results suggest that the presence or absence of a routine distribution in and of itself system shouldn’t influence the decision to account for existing nets in a mass campaign. However, they also indicate that routine distribution through ANC or EPI could aid in maintaining a much younger net crop after a mass distribution, especially if the gap between repeated campaigns is large. Recent work by Okell and colleagues [30] indicates that adding channels which target ITN delivery to age groups at the highest risk of mortality (such as combining infant targeted LLIN delivery with mass campaigns) could produce substantially larger health benefits. The results are shown in Figure 8.

While several studies have documented variations in net durability under different climatic conditions [29,31,32], direct comparisons of net durability between arid, semi-arid, and tropical environments and how these variations influence net effectiveness and vector and malaria transmission dynamics over time is lacking [33]. Further, there is a paucity of research on the role of textile choice (e.g. cotton, which deteriorates faster versus polyethylene, which often maintains integrity longer than cotton) and human behavior (e.g. net use frequency and duration) as it relates to the physical deterioration of nets as they age; some studies have however documented decreased protection as a function of increased washing frequency over time [12,34], the development of broken seams and large holes as the net ages [35,36], and decreased residual efficacy of the insecticide [37], presumably as a function of use, the environment, and maintenance. From an epidemiological perspective however, it appears that the condition of the net is less important than the amount and effectiveness of insecticide present, with intact nets (e.g. no holes) with high levels of insecticide likely providing the most protection against malaria transmission [35], at least in the absence of widespread vector resistance.

A second mechanism by which age influences net effectiveness via net condition relates to use; data from Luangwa District in Zambia [38] suggest that the age (and by proxy, condition, as older nets are often more deteriorated than newer nets) of nets influenced whether they were used, which would in turn influence both the individual and community-level protection. Other reports suggest that individuals prefer clean nets, irrespective of whether continued washing reduces the effectiveness [34]; as older nets are more likely to be more damaged than new nets, the age of the net could reduce overall use [36]. Mathematical models have corroborated the relationships between increasing age of nets and their respective decreasing protective effects [4,33,39,40]. Although current data and models provide a useful mechanism for discussions around optimal methods for measuring net decay and effective usefulness to inform net replacement programs, it is clear that not all LLINs will remain effective under programmatic conditions, with failure beginning immediately after distribution and individual nets remaining effective for varying periods, and that the effect of net condition on malaria transmission likely varies across both time and space.

Additionally, the epidemiological usefulness of LLINs will be influenced by the presence of insecticide resistance and baseline transmission intensity. Thus, the implications of choosing to account for existing nets and thereby leave some older LLINs in the field may have implications for the development of *de novo* insecticide resistance mutations and for selection of existing resistance genes. Additionally the effects of accounting for nets and thus having an older net crop may vary across settings of varied baseline transmission intensity. To determine the implications of each of these with relation to net age, the following questions should be considered: is accounting for existing nets more likely to increase the risk of initial development or spread of insecticide resistance mutations? And, does pre-intervention transmission intensity influence the effectiveness of LLINs as they age?

The effect of insecticide resistance [both metabolic resistance – mediated by a change in the enzymes that detoxify insecticides, and target site resistance – mediated by a change in the molecule the insecticide normally attacks: *e.g.* the *knock-down-resistance* (*kdr*) gene] on field effectiveness of LLINs remains unclear though studies have shown reduced efficacy in experimental huts [41]. It is worth noting that resistance can also result from other mechanisms including behavioral ones, but that the epidemiological importance of these mechanisms is also poorly understood. Similarly, it is unknown what level of insect mortality is needed to confer full epidemiological protection under programmatic conditions, and some studies have shown that the phenotypic expression of resistance in malaria transmitting (older) mosquitoes may be less prominent than in younger genotypically resistant mosquitoes [42,43]. A second theory suggests that any level of insecticide treatment would be beneficial in areas with high frequency of *kdr* resistance genes (such as in West Africa), in terms of reducing vector density, sporozoite rates, and malaria incidence [44] due to the low levels of irritability in mosquitoes carrying the *kdr* gene; this low irritability allows the mosquito to remain in contact with the insecticide long enough to pick up a lethal dose, resulting in substantial reductions in biting rates, sporozoite rates, and parasite transmission even in the presence of *kdr* resistance [45].

It is well established that at high coverage levels (i.e. ≥80% of households possessing at least one treated net) the insecticide in LLINs kills mosquitoes that seek a blood meal thereby reducing vector indoor resting densities by as much as 90% [46]; if the person under the net is already infected with the malaria parasite, the ITN also prevents them from infecting mosquitoes and leading to further transmission. Based on these mechanisms, there is robust evidence from trials that LLINs at relatively high population coverage levels provide community-wide protection, whereby unprotected individuals within or in proximity to high ITN coverage areas are conferred protection from infectious bites [22,44,47]. Models of malaria transmission and ITNs also support this evidence of a mass community effect of ITNs [4,39,48]. Thus moving towards universal coverage at both inter- and intra-household levels (e.g. > 80% of houses covered and >80% of people in houses are under nets) likely provides the strongest protection against transmission.

A conservative approach to limiting the epidemiological implications of accounting for older nets would be to only account for nets below a certain age, for instance to only account for nets below two years of age at the time of replacement. Such an approach could easily be simulated in the model proposed here by limiting the numbers of LLINs in future coverage estimates and gap estimates to only include those less than two years of age.

### General limitations

While stochastic simulation can be used to capture the uncertainty inherent in the parameterization of models such as this, several limitations should be noted. Such models cannot account for exogenous factors including maturational trends (*e.g*. long terms trends in improvements of LLIN manufacture leading to longer average LLIN lifetimes), and the possibility of unaccounted for events such as large scale flooding leading to mass losses of previously functional LLINs. Further such models are subject to parameter uncertainty and model uncertainty. In this exercise, parameter uncertainty around the feasibility of identifying LLINs during household registration is of paramount importance. Model uncertainty could arise from any number of factors in the model specification, but special note should be taken of the fact that covariance between the different stochastic parameters in simulations has not been modeled. This could lead to bias in estimation of the probability of a given coverage arising if the stochastic parameters are in fact correlated. Finally, the NetCALC algorithm functions at a population level to estimate coverage. It thus assumes inter-household net re-distribution. This means that households that receive too many LLINs will redistribute them to other households, the extent to which this happens in practice is not known. This potentially limits the ability of the NetCALC to model strategies where nets are delivered to specific households in excess where net re-distribution does not occur.

## Conclusions

It is clear from this study that attempting to account for nets rather than conduct a blanket distribution should be the strategy of choice only when select conditions are met. Ultimately the threshold in coverage above which it is useful to consider accounting for nets cannot be established universally, but it can be inferred that if feasibility estimates remain as low as noted anecdotally, any cost-savings achieved through such an approach are likely to be small unless household ownership exceeds 40%. Though in most cases accounting for existing nets will not greatly affect the mean age of net crop, a working definition of a useful LLIN will be necessary in all such campaigns, and as such accounting only for nets less than two years of age should be considered. This would mean that only if coverage with nets of less than two years of age exceeds 40% in the last pre-distribution survey should accounting for existing nets even be considered. At coverage above this level it is recommended that programs make a concrete attempt to estimate the feasibility, additional cost and potential epidemiological implications of accounting for existing nets including accounting for the time to the planned distribution. It should be noted however, that any actual attempt to adjust total net procurement or distribution downward by accounting for existing nets carries with it an increased risk of failing to supply enough nets to meet coverage goals, and of reducing the overall effectiveness of the achieved coverage because of the remaining, older potentially less effective nets.

It is probably not possible to set one level which would be thought to represent high enough coverage to justify accounting for existing nets in all situations, given that time since coverage surveys will vary, existence and functionality of routine systems will vary, and the potential duration of epidemiological effects of mass distributions may vary from setting to setting. There remains limited information to base estimates of the additional cost or operational feasibility of accounting for existing nets in mass distribution exercises. While we have not conducted an analysis of the expected value of perfect information, it is clear from the modeling results presented here that two areas of research will be of the most value for garnering further information: one is the feasibility of identifying useful nets at the household level, and the second is the cost of doing so. Both of these areas may have significant impact on the decision and information regarding either from well-documented studies is essentially non-existent. Though our models showed little impact on average net age when nets were accounted for vs. unaccounted for, ultimately the epidemiological implications of adopting either approach cannot be known at this time. Further, given the work of Briet and colleagues [17] it is likely that such a choice might have different implications in different transmission settings, though it is likely that the negative implications of continuing to use aging nets will be more substantial in higher transmission areas.

What is feasible will additionally depend on acceptance by administrative and political leaders and their communities, as well as on practical planning issues such as confusion about distribution rules and registration objectives.

## Competing interests

The authors declare they have no competing interests.

## Author’s contributions

JY, ML and KK conceived of and designed the study, JY contributed to the development of the computer simulation package, conducted simulations and drafted the manuscript, TE, JK and AB aided in the design of the study, conducted the literature review and aided in the drafting of the manuscript, RY aided in the design of the study, the development of the computer simulation package and the drafting of the manuscript. All authors read and approved the final manuscript.

## Supplementary Material

Additional file 1 Implementing the software and a worked example. Click here for file

Additional file 2 [R] package which contains implementation code for the net coverage model described in the paper. Click here for file

Additional file 3: Figure S2 Results of worked example. Legend: Results of 100 simulation runs for a population of one million persons with an average household size of 5.5 and a population growth rate of 3% per year, an initial LLIN coverage of approximately 40% of households having access to at least one LLIN estimated through an unbiased population based survey with a sample size of 3,000, and a campaign three years after the household survey intended to reach approximately 80% LLIN of households owning at least one LLIN. Light center line shows mean of all simulations and dark outer lines represent 80% confidence bands (region within which 80% of simulation results lie). Click here for file

Additional file 4 Details of model structure and [R] code. Click here for file

Additional file 5: Figure S1 Over-dispersion parameter estimated from DHS and MIS data vs. average number of ITN per household. Legend: Thirty-three DHS and MIS surveys are included and a non-linear regression line shown in red for illustrative purposes only. Click here for file
