# Supplementary material for: Planning long lasting insecticide treated net campaigns: should households’ existing nets be taken into account?
Source: Parasit Vectors. 2013 Jun 14;6:174. doi: 10.1186/1756-3305-6-174 (PMC3689647; doi:10.1186/1756-3305-6-174)
Supplement: Additional file 1 — Implementing the software and a worked example. [file 1756-3305-6-174-S1.docx]

**Additional File 2. Implementing the software and a worked example**

This section presents a straightforward description of how to implement the software used in this paper. It also presents an example of a simulation carried out from initial inputs through plotted results and summary statistics.

In order to implement the software utilized in this paper it is first necessary to install the [R] software on your computer. [R] is an open access software for statistical computing which is available for download from the [R] website [http://www.r-project.org](http://www.r-project.org/).

The next necessary step is to load the implementation code into the working memory of the [R] software. This can be done by installing then loading the NetCalcSimulation package, which is available from the corresponding author. The code for installing and loading the package is included below:

install.packages(″.../NetCalc_0.2.tar.gz″, repos=NULL, type=”source”)

library(NetCalc)

Once installation is complete, subsequent use will only require the user to load the package upon each restart of [R]. This can be done using the library command as shown above.

With the NetCalcSimulation software loaded into memory, simulations can be implemented by assigning initial parameter values and calling for simulations. The following section demonstrates the simulation of a mass campaign three years after a coverage survey was conducted.

**Parameter initialization:**

**Pop** the population of interest at time zero (or the start of simulation)

**Popgrowth** the growth rate of the population in percentage (e.g. 0.03 for 3% growth)

**k** a vector containing the lower and upper bound of the k parameter in the smooth compact decay function. In the paper above c(16,18) was used to encapsulate the range of k values covering mean LLIN lifetimes from 2 to 4 years.

**L** a vector containing the lower and upper bounds of the L parameter in the smooth compact decay function. In the paper above c(9.8,20.7) was used to encapsulate the range of L values covering mean LLIN lifetimes from 2 to 4 years.

**LLINdist1** a vector containing the number of LLINs to be distributed through a specific distribution channel in each of the upcoming 20 years. Defaults to c(rep(0, times=20)) (e.g. c(25,450,1000,rep(0, times=17))

**LLINdist2** a vector containing the number of LLINs to be distributed through a specific distribution channel in each of the upcoming 20 years. Defaults to c(rep(0, times=20))

**LLINdist3** a vector containing the number of LLINs to be distributed through a specific distribution channel in each of the upcoming 20 years. Defaults to c(rep(0, times=20))

**LLINdist4** a vector containing the number of LLINs to be distributed through a specific distribution channel in each of the upcoming 20 years. Defaults to c(rep(0, times=20))

**LLINdist5** a vector containing the number of LLINs to be distributed through a specific distribution channel in each of the upcoming 20 years. Defaults to c(rep(0, times=20))

**Init.coverage** a vector containing the average number of LLINs available per household by age at the start of the simulation. Defaults to c(rep(0, times=20)).

**Init.size** a vector of size parameters for the negative binomial distributions used to model the estimated average number of LLINs of a given age per household in stochastic simulation of coverage of LLINs derived from survey data. Defaults to c(rep(0, times=20)).

**Init.samplesize** the sample size (number of households) of the survey used to estimate initial

LLIN coverage levels

**HHsize** average household size

**coverage** a logical parameter which is set to TRUE if the user desires the model output to be in terms of household ownership of LLINs or FALSE if the user desires the model output to be a matrix containing the number of LLINs available in each year of simulation by each year of net age. Defaults to TRUE.

**times** the number of simulation runs to be executed. Defaults to 100.

The following script runs a set of 100 simulation runs for a population of one million persons with an average household size of 5.5 and a population growth rate of 3% per year, an initial LLIN coverage of approximately 40% of households having access to at least one LLIN estimated through an unbiased population based survey with a sample size of 3,000, and a campaign three years after the household survey intended to reach approximately 80% LLIN of households owning at least one LLIN.

These commands set the initial simulation parameters:

####Below is a list of initialization parameters

Pop<-1000000

Popgrowth<-0.03

k<-c(16,18)

L<-c(9.8,20.7)

LLINdist1 <- c(0,0,440000,rep(0,times=17))

Init.coverage<-c(1,0.5,0.5,0.5,rep(0, time=16))

Init.size<-c(1,1,1,1, rep(0,times=16))

Init.samplesize<-3000

HHsize<-5.5

The following command runs 100 simulations:

Results<-NetCalcSimulation(Pop=Pop, Popgrowth=Popgrowth, k=k, L=L, LLINdist1=LLINdist1, Init.coverage=Init.coverage,Init.size=Init.size, Init.samplesize=Init.samplesize, HHsize=HHsize)

Results can now be summarized and visualized using the following commands:

mean.coverage = colMeans(Results)

plot(type=’l’, mean.coverage, col="#06060015", ylab="% HH with at least 1 LLIN", xlab="Year", ylim=c(0,1.0))

In order to estimate and plot the proportion of simulations which fall between specified intervals use the following script which presents the intervals between which 80% of simulations fall:

### Calculate quantiles across the matrix

CIs = apply(Results, 2, quantile, probs = c(0.1, 0.90))

lines(CIs[1,],type=’l’)

lines(CIs[2,],type=’l’)

Implementation of the above script will result in a figure similar to Figure A2 and similar to the example from the [R] package.
