# Supplementary figures and images for: Planning long lasting insecticide treated net campaigns: should households’ existing nets be taken into account?
Source: Parasit Vectors. 2013 Jun 14;6:174. doi: 10.1186/1756-3305-6-174 (PMC3689647; doi:10.1186/1756-3305-6-174)

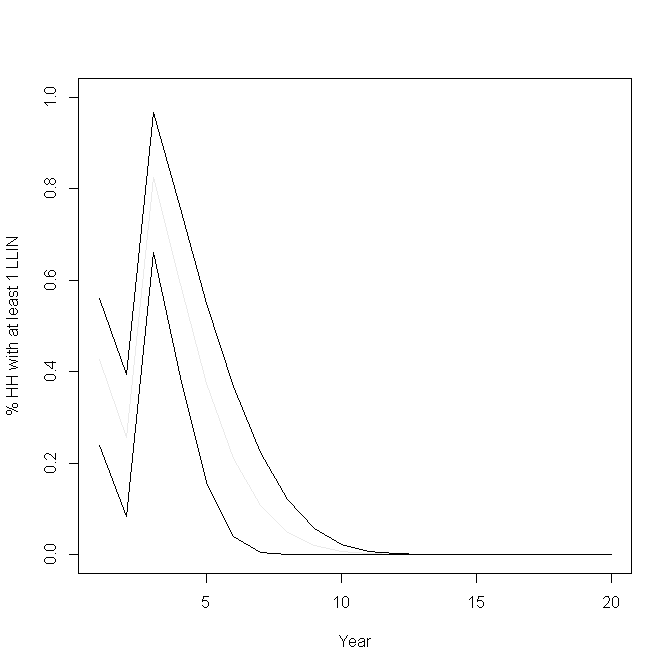

Supplement: Additional file 3: Figure S2 — Results of worked example. Legend: Results of 100 simulation runs for a population of one million persons with an average household size of 5.5 and a population growth rate of 3% per year, an initial LLIN coverage of approximately 40% of households having access to at least one LLIN estimated through an unbiased population based survey with a sample size of 3,000, and a campaign three years after the household survey intended to reach approximately 80% LLIN of households owning at least one LLIN. Light center line shows mean of all simulations and dark outer lines represent 80% confidence bands (region within which 80% of simulation results lie). [file 1756-3305-6-174-S3.png]

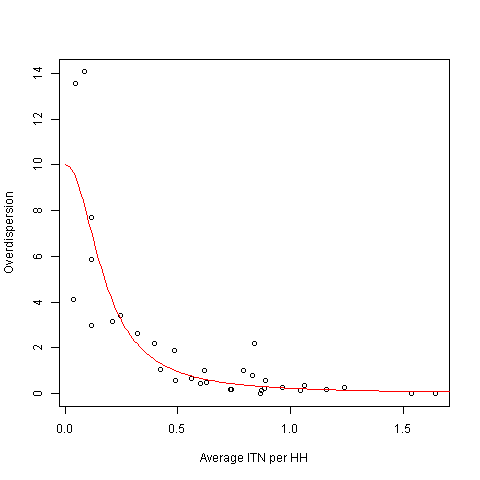

Supplement: Additional file 5: Figure S1 — Over-dispersion parameter estimated from DHS and MIS data vs. average number of ITN per household. Legend: Thirty-three DHS and MIS surveys are included and a non-linear regression line shown in red for illustrative purposes only. [file 1756-3305-6-174-S5.png]
