# Supplementary material for: Planning long lasting insecticide treated net campaigns: should households’ existing nets be taken into account?
Source: Parasit Vectors. 2013 Jun 14;6:174. doi: 10.1186/1756-3305-6-174 (PMC3689647; doi:10.1186/1756-3305-6-174)
Supplement: Additional file 4 — Details of model structure and [R] code. [file 1756-3305-6-174-S4.docx]

**Additional File 1. Details of model structure and [R] code.**

To simulate the effect of sampling error on the decision whether to account for LLINs in planning a “top-up” campaign, we modeled the existing numbers of nets by sampling from a negative binomial distribution of net ownership of a given age within households at the time of the survey. This was simulated and parameterized according to the following method:

$$Avg_{Age}=\frac{\sum_{1}^{SS} N_{a_{SS}}\sim negativebinomial\left(N_{\hat{a}},size\right)}{SS}$$

where *N_a_* is the number of LLINs of a given age for a simulated household; size is the over dispersion parameter; *SS* is the number of households sampled in the survey; and *N_â_* is the estimated average number of nets of a given age per household as determined from survey data. The total number of nets assumed to be present in each simulation was determined by resampling from this distribution “*SS”* number of times, and averaging the results, then scaling up by the number of households in the simulation. Thirty three MIS and DHS surveys were used to generate estimates of the over-dispersion parameter. The over-dispersion parameter for the estimated average number of LLINs available per HH in the country was determined to be near to one in the coverage ranges of greatest interest for purposes of this paper (Figure A.1). These simulations resulted in a vector of numbers of nets actually available in each survey, which were input into simulations.

Time since a survey may also impact decisions around existing coverage levels. Nets decay, are lost, disposed of, repurposed or otherwise cease to exist over time. We incorporated this phenomenon by allowing survey data to measure both the age of existing nets and at varying points before the distribution of interest was simulated. We used methods from the NetCALC model to simulate decay of LLINs.

Lifetimes of LLINs were modeled using the following “smooth-compact” decay function fit by Nakul Chitnis to Albert Killian’s LLIN retention data [16]. This is the function used in the NetCALC software.

The function predicts the proportion of LLINs available *t* years following a distribution. For a half-life of three years (50% of LLINs remaining 3 years after their initial distribution), this function is parameterized with *k*=17 and *L*=15.1. We used a uniform distribution to parameterize a range of decay functions by drawing a decay parameterization at random from the following distributions. *k~* uniform(16,18) and *L~* uniform (9.8,20.7), which cover the range of half-lives from 2 to 4 years. For each simulation, a random combination of the two parameters was chosen and used to simulate the lifetimes of all nets in the simulation.

Additionally, expected coverage after a survey can be affected by distributions of LLINs between the survey estimate and the time of the planned mass distribution under consideration. We incorporated these estimates by inputting new LLINs into the simulation in the following manner. We only considered routine distribution systems here as it was assumed that such a decision around whether or not to account for existing LLINs would be at the time of the first mass distribution after a coverage survey, rather than many years or many mass distributions later. As many African countries have used existing antenatal care (ANC) and expanded program on immunization (EPI) platforms to distribute LLINs, we based our routine net distributions in simulations accordingly.

The number of LLINs distributed through ANC or EPI was modeled deterministically according to the following assumptions. We chose to set the total initial population in our simulations to one million persons. The relative population size is unimportant as the model estimates identical coverage for any population size (the total population size functions only to scale the results and as such one million is a convenient number). Demographic estimates were chosen to be typical for a sub-Saharan African country and were based on UN population estimates (United Nations Population Division World Population Prospects Database (*http://esa.un.org/unpd/wpp/index.htm*). The population structure was set with 4% of the population assumed to be pregnant at any given time, and 17% of the population consisting of children under the age of five. The growth rate of the population was assumed to be 3% per year, a value in the upper range of growth estimates for most SSA countries, and was assumed to be demographically stable, such that in each year the proportion of women who were pregnant remained constant, the proportion of the population that consisted of children under five years of age remained constant and the absolute numbers in each group grew in proportion to the total population size. The average household size was assumed to be 5.5 persons, a mid-line value for SSA; in these simulations this translates to approximately 181,820 households [50].

The numbers of pregnant women and children who are eligible for either ANC or EPI services were estimated based on the following assumptions. At any given time 4% (or approximately 40,000 women) were assumed to be pregnant; given an assumption of a 9 month term, over the course of the year approximately 5% (4%*1.25) of the population was considered eligible for ANC services. The rate of success of such services of delivering LLINs to eligible women was varied between 0% and 100%, incorporating both the coverage of services and the success rate of services at delivering an LLIN given that a woman attended ANC. (These numbers could be varied separately but this method gives a reasonable summary and can be used to cover all possible combinations of two separate probabilities). For simulations where a working ANC distribution system was in place, we assumed that 80% of all eligible women would receive an LLIN through such a distribution mechanism each year. This is roughly equivalent to a system where 90% of eligible women access ANC services each year and that the services distribute LLINs to 90% of women who accessed services. The estimates translate to 4% of the population receiving LLINs through ANC services each year or approximately 40,000 LLINs. For EPI, we assumed that 3.7% of the population was under one year of age in each given year and that all of these children would be eligible for EPI services. The rate at which such services were accessed and successfully delivered LLINs each year was varied from 0% to 100%. For estimates of a functioning EPI distribution system we simulated a system in which 90% of infants (under one year of age) would access routine EPI services each year and the services would provide a LLIN to these children successfully 90% of the time. This is again equivalent to 80% of eligible (infant) children receiving an LLIN each year. This leads to an estimate of 29,600 LLINs distributed annually through such an EPI distribution mechanism. Around 69,600 LLINs might be distributed each year through a combined ANC-EPI mechanism. If 100% of the target population was successfully reached each year, approximately 87,000 LLINs could be distributed each year through such mechanisms. All LLIN distribution mechanisms were assumed to grow in numbers of nets distributed each year in proportion to target population growth.
